# Unique roles of tryptophanyl-tRNA synthetase in immune control and its therapeutic implications

**DOI:** 10.1038/s12276-018-0196-9

**Published:** 2019-01-07

**Authors:** Mirim Jin

**Affiliations:** 1 Department of Microbiology, College of Medicine, Gachon University, Incheon, Korea; 2 Department of Health Science and Technology, GAIHST, Gachon University, Incheon, Korea

**Keywords:** Innate immunity, Infection

## Abstract

Tryptophanyl tRNA synthetase (WRS) is an essential enzyme as it catalyzes the ligation of tryptophan to its cognate tRNA during translation. Interestingly, mammalian WRS has evolved to acquire domains or motifs for novel functions beyond protein synthesis; WRS can also further expand its functions via alternative splicing and proteolytic cleavage. WRS is localized not only to the nucleus but also to the extracellular space, playing a key role in innate immunity, angiogenesis, and IFN-γ signaling. In addition, the expression of WRS varies significantly in different tissues and pathological states, implying that it plays unique roles in physiological homeostasis and immune defense. This review addresses the current knowledge regarding the evolution, structural features, and context-dependent functions of WRS, particularly focusing on its roles in immune regulation.

## Protein synthesis: An unexpected link with immune regulation

Targeting tryptophanyl tRNA synthetase (WRS), an evolutionarily conserved enzyme involved in protein synthesis, could be an effective strategy for modulating the immune system. In addition to helping translate mRNA into amino acid sequences in cytoplasm, human WRS can be secreted and activate immune responses against invading pathogens. Mirim Jin at Gachon University, Incheon, South Korea, reviews recent studies on the structure, expression pattern and functions of WRS other than protein synthesis. High levels of WRS protein have been found in patients with sepsis and autoimmune diseases suggesting that inhibiting WRS could be a potential therapeutic approach for treating these conditions. Further research into WRS will shed light not only on how it regulates the immune system, but also on how it exerts other reported effects on blood vessel formation and cell migration.

## Introduction

Aminoacyl-tRNA synthetases (ARSs) are essential housekeeping enzymes that participate in the translation of mRNAs^1^. ARSs are thought to have emerged at the time of the last universal common ancestor and are distributed across all taxa^2^. The common aminoacylation reaction, which attaches an amino acid to its cognate tRNA, is conserved and proceeds in two steps. First, an amino acid and adenosine triphosphate (ATP), as an energy source, bind the active site of the enzyme and form an aminoacyl adenylate intermediate (amino acid-AMP); second, the adenosine monophosphate (AMP) is displaced by its cognate tRNA, resulting in the covalent linkage of the specific amino acid to one of the ribose 3′-OH moieties of a set of tRNA isoacceptors^3–7^. The 20 ARSs (1 for each of the 20 amino acids) are naturally categorized into two classes (I and II) based on the structure of the ancestral catalytic core, chemical properties, and consensus sequences^8^. Most Class I ARSs (except the Ic subclass) are monomeric, while Class II ARSs are multimeric^9^. Each class can be further divided into subclasses based on their unique organization of conserved structural motifs, anticodon-binding domain characteristics, and mechanical properties. The catalytic domain of all Class I ARSs has a Rossmann Fold (RF) containing the dinucleotide-binding domain; this domain carries out the aminoacylation reaction and is located at or near the N-terminus. Classically, this domain features a five-stranded parallel β-sheet connected by α-helices. Three subclasses within Class I are designated as follows: Ia, for hydrophobic amino acids (Ile, Leu, and Val), sulfur-containing amino acids (Met and Cys), and Arg; Ib, for charged amino acids (Glu and Lys) and Gln; and Ic, for aromatic amino acids, including tyrosine and tryptophan. Class II is characterized by a seven-stranded β-sheet with flanking α-helices, and it is also divided into subclasses: Class IIa, which recognizes groups with chemically similar side chains, including aliphatic (Ala and Pro) and polar (Ser, Thr, Pro, and His) amino acids and Gly; class IIb, which recognizes the charged side chains of Asp and Lys and Asn; and class IIc, which recognizes the aromatic amino acid Phe^4,9,10^.

**Fig. 1:**
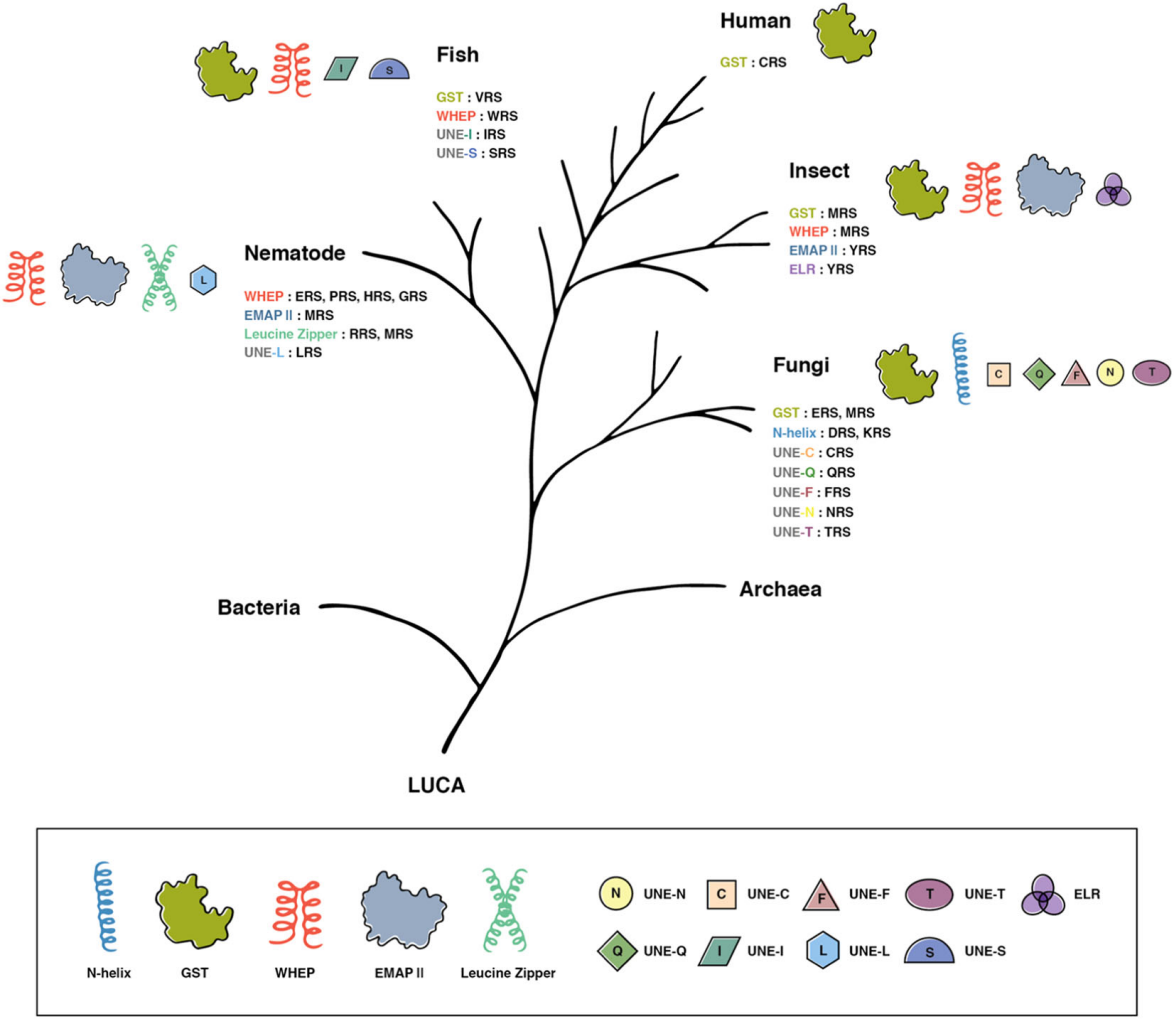
Newly acquired domains and motifs in ARSs during the evolutionary process. During evolution, aminoacyl-tRNA synthetase have acquired new domains, allowing for an increase in the complexity of organisms in a particular phylogenetic group. Importantly, with the exception of MRS, these domains or motifs have been irreversibly retained by their respective tRNA synthetase until the evolution of humans. As a result, each aminoacyl-tRNA synthetase in humans has all of the newly acquired domains. Each of these domains imparts specific new functions that are more complex than simple aminoacylation, which was the original function of these enzymes. The model species used are: *Homo sapiens*, *Danio rerio*, *Drosophila melanogaster*, *Caenorhabditis elegans*, and *Saccharomyces cerevisiae*. LUCA last universal common ancestor, N-helix N-terminal amphiphilic helix, GST glutathione *S*-transferase (GST)-like domain, EMAPII endothelial monocyte-activating polypeptide II

Interestingly, if the evolutionary tree is followed from lower to higher eukaryotes, ARSs can be seen to have progressively and irreversibly added new domains or motifs that have no apparent connection with aminoacylation^11^. Five domains have been found, including the N-terminal amphiphilic helix (N-helix), the glutathione *S*-transferase (GST)-like domain, a helix-turn-helix motif referred to as a WHEP domain (the name is derived from four of the five WHEP containing proteins: *W*RS, histidyl (*H*) tRNA synthetase, glutamyl-prolyl (*EP*)-tRNA synthetase, and methionyl tRNA synthetase), an endothelial monocyte-activating polypeptide II (EMAPII) domain, and a leucine zipper domain^4,11–15^; these domains are shared by more than one ARS. By contrast, eight unique sequence motifs, referred to as UNEs, are specific to only one ARS. The UNEs from cysteinyl tRNA synthetase (CRS), glutaminyl tRNA synthetase (QRS), phenylalanine tRNA synthetase (PRS), aspartidyl tRNA synthetase (DRS), lysyl tRNA synthetase (KRS), asparaginyl tRNA synthetase (NRS), threonyl tRNA synthetase (TRS), and leucyl tRNA synthetase (LRS) are not similar to any of the sequences of bacterial or archaeal tRNA synthetases or to other UNEs (Fig. 1)^11,16^. The occurrence of additional domains or motifs correlates with the emergence of new and more sophisticated species with increasing biological complexity at a correct and timely moment that are capable of performing the novel functions required by developing systems as they evolve^12,17,18^. Furthermore, the structural metamorphosis generated by alternative splicing, proteolysis, and post-translational modification seem to give more diverse functions to ARSs^19–22^. On the other hand, while prokaryotic ARSs exist independently within cells, eukaryotic ARSs have obtained the ability to form a multi-tRNA synthetase complex (MSC)^23,24^. In humans, nine different ARSs and three non-synthetase factors, p43, p38, and p18, are assembled into an MSC, which operates as a functional depot for alternative activities of its members^17,25,26^. Furthermore, most ARSs translocate from the cytoplasm to the nucleus or extracellular space in response to specific stimuli and can participate in various biological processes. Expansions in ARS functions are associated with numerous biological activities, including transcription, translation, cell death and survival, immunity to microbes, chemotaxis and inflammation, angiogenesis, IFN-γ and p53 signaling, mTOR signaling, and tumorigenesis (Table 1)^27–31^.

**Table 1:** Non-canonical functions of the new domains and motifs in selective aminoacyl-tRNA synthetases

Domain	ARS	Acquired function during evolution	References
UNE-S	SRS	Facilitates the translocation of SRS from the cytoplasm into the nucleus to regulate VEGF expression	^16, 86^
UNE-L	LRS	Important for stabilizing RRS and LRS in the MSC	^87^
GST	ERS or EPRS	Included as a component of the MSC	^26^
	MRS	Included as a component of the MSC	^24, 26^
	VRS	Complexes with eEF1H during translation	^21, 88, 89^
	CRS	Complexes with eEF1γ during translation	^90, 91^
EMAP II	YRS	Regulates the cytokine activity of YRS	^92^
Leucine zipper	RRS	Interacts with the MSC	^24, 93^
N-helix	YRS	Increases the affinity of the synthetase for its tRNA in translation	^94, 95^
	DRS	Enhances tRNA-binding affinity	^96– 98^
WHEP	EPRS	Suppresses inflammatory gene expression	^13, 99^
	HRS	Activates chemokine receptors on T-lymphocytes and immature dendritic cells	^100^
	MRS	Translocates to the nucleolus in response to growth factors and enhances rRNA synthesis during transcription	^101^
	GRS	Regulates catalytic efficiency, thermal stability, and structural flexibility	^102^
	WRS	Acts as an endogenous ligand for the TLR4/MD2 complex	^20^
ELR	YRS	Critical for the function of IL-8-like cytokines	^103^

*N-helix* N-terminal amphiphilic helix, *GST* glutathione *S*-transferase (GST)-like domain, *EMAPII* endothelial monocyte-activating polypeptide II

The diverse structural metamorphoses among species, and/or within a species, have evolved by developing differential mechanisms for performing certain functions in immunity. There are a few examples of chemotaxis and angiogenesis: (i) UNE, a N-terminal 80 amino acid domain in the nematode *Brugia malayi* asparaginyl tRNA synthetase (bmNRS), which creates an IL-8 like fold and interacts with the IL-8 receptor, leading to chemotaxis and proangiogenic effects;^32,33^ (ii) an EMAP II-like carboxy-terminal domain and the mini-YRS from human tyrosyl tRNA synthetase (hYRS), which are produced following cleavage by leukocyte elastase and show chemotactic activity. In particular, the mini-YRS induces chemotaxis by binding to the IL-8 type A receptor;^34^ (iii) human WRS (hWRS), which is secreted from monocytes in which the WHEP domain (WRS) interacts with Toll-like receptors (TLR) to stimulate chemokine secretion, and after removal of WHEP, hWRS can exert an angiostatic effect via vascular-endothelial (VE)-cadherin^20,31,35^. Another example is the unique role of the WHEP domain that is associated with the antiviral effect of glutamyl-prolyl-tRNA synthetase (EPRS), which is composed of glutamyl tRNA synthetase (ERS) and prolyl tRNA synthetase (PRS) coupled via a linker containing three WHEP domains. This domain is found on the exterior of the MSC in the cytoplasm. Following viral infection, serine 990, located in the WHEP linker, is phosphorylated to release EPRS from the MSC, after which the amino-terminal domain of EPRS (1–196 aa), which contains a GST-like domain (1–168 aa) and the linker region L1 (168–196 aa), interacts with poly(rC)-binding protein 2 (PCBP2). Consequently, interferon-β production is increased due to the suppression of PCBP2-mediated ubiquitination and degradation of mitochondrial antiviral-signaling protein (MAVS), ultimately leading to the inhibition of viral replication^36^. By acquiring additional, differentiated functions with discrete mechanisms that can differ at specific locations, ARSs are key regulators of physiological homeostasis^18^.

This review focuses on WRS, one of the most extensively studied ARSs. Information regarding its evolution, structural features, and context-dependent biological functions, particularly in immunity, reveals its significant roles in immune regulation and its therapeutic potential.

## Architecture for the catalytic reaction

**Fig. 2:**
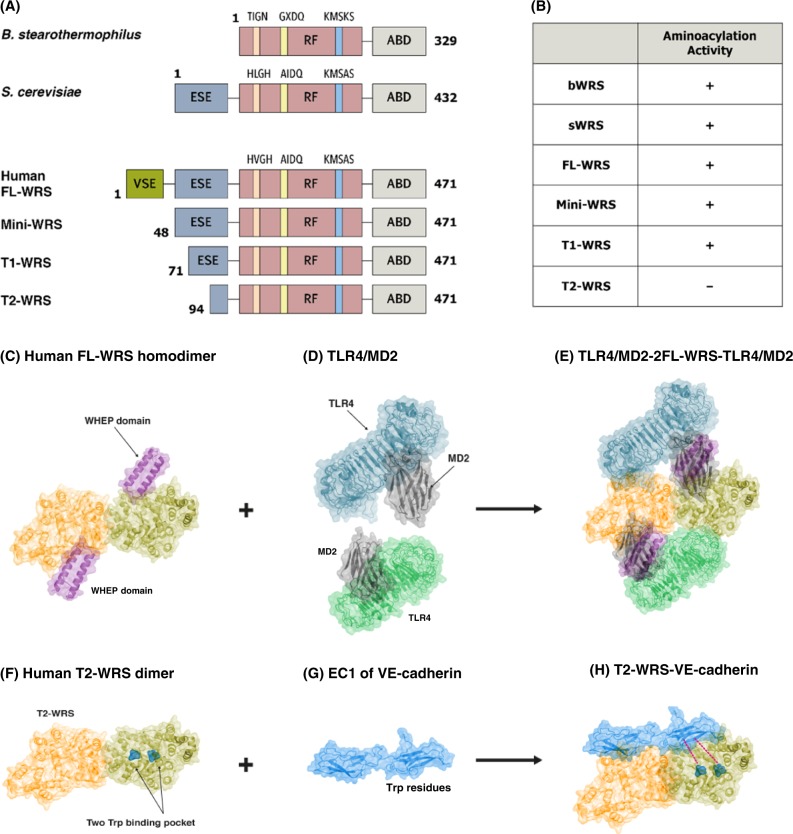
Various structures of WRS and their interactions with specific receptors. **a** Schematic representation of domains in WRS from prokaryotes (*B. stearothermophilus*), lower eukaryotes (*S. cerevisiae*), and higher organisms (*H. sapiens*). The Rossmann Fold (RF) catalytic domain and anticodon-binding domain (ABD) are well conserved in all WRSs. In the RF domain, the three characteristic types of motif have slightly different sequences among species. The eukaryotic-specific extension (ESE) is common in eukaryotic WRSs. Human full-length (FL)-WRS also has the vertebrate-specific extension (VSE) at the N-terminal appended site. Alternative splicing can produce a mini-WRS where a portion of the VSE is truncated. After being secreted into the extracellular space, the T1 and T2-WRSs are generated by proteolytic cleavage, which removes the ESE, including the entire VSE sequence. **b** The aminoacylation activity of the various forms of WRSs in different species. All the WRSs, with the exception of T2-WRS, are capable of aminoacylation. **c**–**h** Representative model of the structural arrangement of WRS and their specific interactions with receptors. FL-WRS (**c**), dimeric human FL-WRS (PDB 1R6T) binding to two TLR4/MD2 heterodimers (PDB 3FXI) through the WHEP domain and the N154 terminus *in trans* (**d**, **e**), T2-WRS (PDB 1O5T) with truncated WHEP entirely cuts off the N-terminus (**f**), interaction of T2-WRS with the EC1 domain in VE-cadherin (PDB 3PPE). Trp residues present in the EC1 domain bind to the Trp binding pocket present in the RF domain of WRS (**g**, **h**)

Specific recognition by tryptophanyl tRNA synthetase (WRS) of its substrates, Trp and tRNA, is critical for maintaining fidelity in protein synthesis. WRS belongs to the Class Ic ARS family, containing an RF domain with two highly conserved signature sequences, namely, KMSKS (Lys-Met-Ser-Lys-Ser) and HIGH (His-Ile-Gly-His); the former contributes to amino acid activation and the latter stabilizes both ATP during amino acid activation and the 3′ end of the tRNA for amino acid transfer^2,37,38^. The C-terminal alpha helical domain is the binding site for the tRNA anticodon. While the WRS from *Bacillus stearothermophilus (*bWRS) requires two domains for the catalytic reaction, eukaryotic WRSs, such as those from yeast and archaea, have acquired an extra N-terminal domain, referred to as a eukaryote-specific extension (ESE), of 100 amino acid residues^2,38^. Furthermore, in vertebrates, including humans, WRS has an N-terminal extension of 154 amino acids (N154) that is composed of the ESE and an additional vertebrate-specific extension (VSE), constituting the full-length (FL)-human WRS comprising 471 amino acids^31,39,40^ (Fig. 2a). Although the reaction catalyzed by WRS occurs through a similar “induced-fit mechanism” and involves a profound reorganization of the RF domain through conformational changes, human WRS uses a more complex catalytic reaction than bWRS^41^. For example, in the aminoacylation reaction, homo-dimeric WRS exhibits “half-site” activity, meaning that only one monomer of the homo-dimeric WRS operates at a time. A structural analysis of the WRS-tryptophan-ATP complex has revealed that one WRS monomer, in the form of a semi-closed KMSAS loop, binds to tryptophan, whereas the other monomer, in the form of a closed KMSAS loop, binds to both tryptophan and ATP^37^. Recognition of Trp induces a conformational change in the AIDQ motif of human WRS to generate a deep pocket for Trp binding and the activation and coupled movement of the N-terminal extension and C-terminal domain, leading to ATP binding of the KMSAS loop in a closed conformation, thereby securing the position of ATP for catalysis and coupling of the C-terminal tRNA-binding domain for Trp transfer^4,37,38,42^.

## Structural metamorphosis for non-catalytic reactions

Undoubtedly, the N-terminal extension domain of human WRS was adopted to undertake non-canonical functions. The addition of the WHEP domain, WRSs occurred at the chordate stage and have been preserved ever since (Fig. 1). Recently, it has been proposed that a dimeric FL-WRS (Fig. 2c) can crosslink with two toll-like receptor 4-myeloid differentiation factor 2 (MD2) (Fig. 2d) heterodimers via N154, where WHEP (from 8–64), located in the N154 regions, is pivotal. WHEP inserts between the TLR4 and MD2 of one heterodimer, with the terminal domain of the N154 region binding the other TLR4 in *trans*, leading to TLR4-MD2 dimerization and macrophage activation (Fig. 2e). By contrast, mini-WRS, which lack parts of the WHEP domain, seldom induces functional dimerization^11,20^. Rather, mini-WRS, a naturally occurring alternative splice variant of WRS that lacks the N-terminal 47 amino acids, and T1- and T2-WRS, which are proteolytic products of secreted FL-WRS and lack significant parts of the WHEP domain, are composed of 71–471 and 94–471 amino acids, respectively, and have anti-angiogenic effects^11,43,44^. For example, after proteolytic cleavage, T2-WRS (Fig. 2f) binds VE-cadherin, an endothelial cell adherens junction molecule. VE-cadherin has two conserved tryptophan residues at positions 2 and 4 of the N-terminal extracellular domain (EC)-1, which is a key determinant for the dimerization of VE-cadherin in preformed blood vessels. In newly generated blood vessels, EC1 in the VE-cadherin monomer (Fig. 2g) is exposed owing to endothelial cell budding. Similar to other ARSs, human WRS has developed a tryptophan-binding pocket as a specific active site^27,39,43,45,46^. After proteolytic cleavage of the N-terminal extension covering the binding pocket, the tryptophan-binding sites are exposed, and the two proteins bind via the tryptophan residues in EC1 (Fig. 2h)^47^. Therefore, it is reasonable that human FL-WRS, with a completely preserved N-terminal extension, does not exert angiostatic effects^48,49^. N154 in human WRS has probably evolved to achieve a non-catalytic multi-function while simultaneously contributing to catalytic activity based on the fact that T2-WRS cannot perform aminoacylation because of the lack of the N-terminal extension domain participating in the reaction, which mini-WRS can perform (Fig. 2b)^44^.

## WRS signaling and clinical relevance

### Extracellular signaling of secreted WRS

**Fig. 3:**
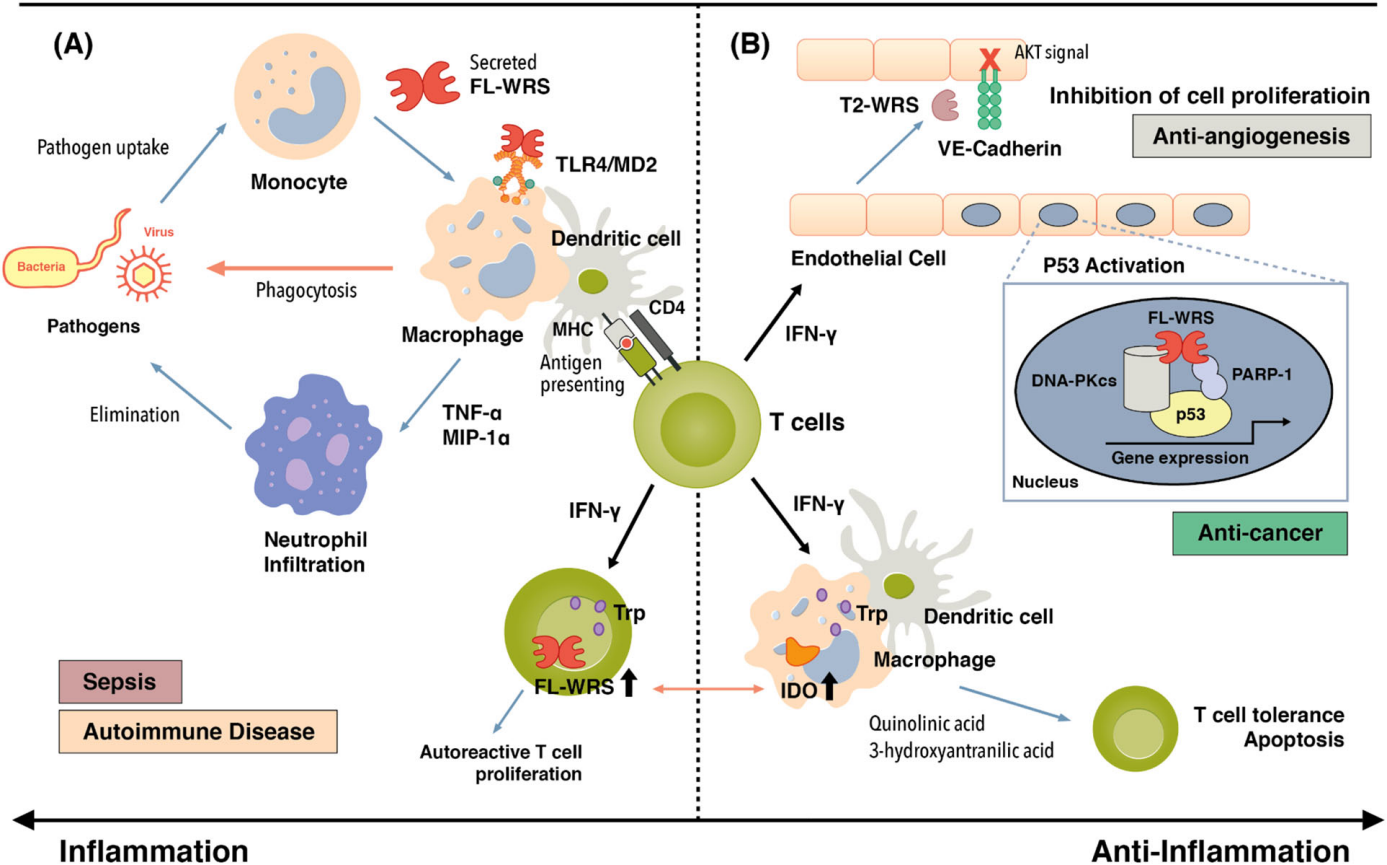
Roles of WRS in “Yin-Yang” immune regulation and its relation to immune diseases. **a** Upon infection, FL-WRS is rapidly secreted from monocytes to activate macrophages via TLR4/MD2, which induces innate immune responses, including chemokine production, neutrophil infiltration, and increased phagocytosis, eventually leading to pathogen elimination. High levels of WRS probably provoke an acute hyper-inflammation in sepsis. Antigen presentation to T cells by dendritic cells induces IFN-γ production, leading to the increased expression of WRS. Overexpression of WRS in T cells is able to reserve Trp, which is essential for cell activation and proliferation and may be involved in the development of autoreactive T cells in autoimmune diseases. **b** IFN-γ stimulation not only increases WRS secretion but also induces the translocation of WRS into the nucleus. Secreted WRS is proteolytically processed to form T2-WRS, which binds to VE-cadherin and inhibits angiogenesis. Nuclear FL-WRS enhances anti-angiogenesis by regulating cell growth through the phosphorylation of p53, further implying a role for WRS as an anti-cancer agent. Furthermore, an imbalanced activation of indoleamine 2,3-dioxygenase (IDO) relative to WRS in antigen-presenting cells (APCs), including macrophages and dendritic cells, induces an immunosuppressive state, inhibiting the proliferation of immune cells, including T cells, and possibly causing these cells to become tolerant or apoptotic

WRS is secreted into the extracellular space in response to certain stimuli. For example, upon pathogenic infection, but prior to tumor necrosis factor-α (TNF-α) production, WRS is rapidly secreted from monocytes without de novo synthesis, although the mechanism of secretion is not completely known. The secreted FL-WRS, but not mini-WRS, interacts with TLR2 and/or TLR4-MD on macrophages, leading to the activation of innate immune responses, in which TNF-α and chemokine production, neutrophil infiltration, and increased phagocytosis are prominent. These responses eliminate invading pathogens in the very early phase of infection, implying that there is a crucial role for FL-WRS as an endogenous ligand of human TLR2/4 in countering infections and immune regulation (Fig. 3a)^20,50^. In support of this hypothesis, compared with healthy subjects, high levels of WRS are consistently detected in the serum of critically ill patients with sepsis, a potentially lethal complication of a severe infection. Recent preliminary data have shown that there is a significant positive correlation among WRS levels and sepsis severity, the sepsis-associated organ failure assessment (SOFA) score, and deaths (unpublished data); the data therefore suggest a pivotal role for WRS in sepsis pathophysiology.

By contrast, when endothelial cells are stimulated with IFN-γ, an angiostatic cytokine, the expressed WRS forms a tertiary complex with annexin II and S100A10, which regulate exocytosis. A small fraction of the complexed WRS can become dissociated from annexin II-S100A10 and be secreted from cells^51^. This secretion does not seem to proceed through the classical secretion pathways involving the Golgi, as it is not blocked by treatment with brefeldin A or A23187, inhibitors of ER-Golgi-transport and calcium-dependent exocytosis, respectively^51,52^. After secretion, FL-WRS is cleaved by plasmin and/or elastases, which are both critical proteases involved in angiogenesis^53^. T2-WRS potently inhibits new angiogenesis, acting via VE-cadherin, a critical player in angiogenesis and vascular permeability at the intracellular junction between endothelial cells^31,35,39,48,54^. This T2-WRS/VE-cadherin interaction prevents the activation of VEGFR and subsequent ERK-mediated signaling, which suppress endothelial migration and proliferation (Fig. 3b)^48,55,56^.

### Intracellular signaling of WRS

Since WRS is a housekeeping enzyme, its overexpression in various cells and tissues under diverse physiological environments is a rather unexpected finding^57–59^. With respect to development and aging, WRS expression is increased both in the developing salivary grand of *Drosophila* and in aged human epidermis^60^. WRS mRNA levels have been shown to be increased for a specific time during the differentiation of human monocyte-derived macrophage and dendritic cells^61,62^. Furthermore, WRS is highly expressed not only in cells infected with human cytomegalovirus and hepatitis B virus but also in mouse intestines infected with *Cholera vibrio*^63–65^. However, the reasons for this WRS overexpression and the underlying mechanisms remain unclear.

Endothelial WRS mRNA expression can also be increased in response to IFN-γ, which is mediated by transcription factor binding to gene promoters, including the gamma-activated sequence and the interferon-sensitive response element in STAT-1 and IFN regulatory factor, respectively. WRS can translocate into the nucleus, using a potential nuclear localization sequence proximal to the C-terminus^27^. In the absence of nuclear WRS, DNA-PKcs (the catalytic subunit of DNA dependent protein kinase) is independently linked to poly [ADP-ribose] polymerase 1 (PARP-1), with Ku70/80 serving as a bridge between the two. Ku70/80 binding to both DNA-PKcs and PARP-1 orients the C-terminal domain of PARP-1 to allow for its phosphorylation by DNA-PKcs. In this state, PARP-1 cannot PARylate DNA-PKcs. However, nuclear WRS can displace Ku70/80 and bind to both DNA-PKcs and PARP-1. The WHEP domain of WRS bridges the C-terminal kinase domain of DNA-PKcs to the N-terminal domain of PARP-1, where WRS enables Trp-AMP, an intermediate amino-adenylate product, to occupy the active site. When Trp-AMP occupies the active site of WRS, the WHEP domain opens and is available for interacting with DNA-PKcs and PARP-1, thus stimulating the PARylation of DNA-PKcs by PARP-1. Finally, the PARylated DNA-PKcs then phosphorylates p53, leading to p53-driven anti-proliferative effects and senescence^27^. These findings imply that the functions of nuclear WRS may concur with those of extracellular WRS as an angiostatic cytokine, consistent with the well-known functional integration of IFN-γ signaling and p53 activation. This furthermore suggests roles for WRS in cell death or cancer (Fig. 3b)^66^.

Tryptophan metabolism is closely linked to IFN-γ-mediated immune regulation, which induces a dual effect on its metabolism. Firstly, there is an increase in the rate of degradation of Trp by the indoleamine 2,3-dioxygenase (IDO) pathway, where IDO is strongly increased in macrophages and dendritic cells. The resulting depletion of Trp levels represses immune cell activation and proliferation in the microenvironment^67–73^. In addition, the products of Trp catabolism (quinolinic acid and 3-hydroxyantranilic acid) can induce T cell apoptosis, leading to immune tolerance. Secondly, the accumulation of tryptophan into the Trp–tRNA complexes available for protein synthesis provides a protective mechanism for immune cells (Fig. 3b)^74,75^. Treatment of cytotoxic T lymphocyte antigen-4 (CTLA-4), a negative regulator of T cell activation, with human PBMCs induced concomitant increased expression of both IDO and WRS in CD4+ T cells, which prevents CD4+ T cell activation through an IDO-dependent mechanism. Moreover, CD8+ T cells only showed increased WRS expression, not IDO, and were able to maintain their activation status via the Flu antigen, which is unaffected by CTLA-4 pretreatment, rendering WRS a Trp reservoir for protein synthesis^76,77^. In addition, given that immune cells possess many tryptophan-rich proteins compared with proteins in general, such as the human major histocompatibility complex antigens, complement factor B, and β-2 microglobulin, which are known to be induced by IFN-γ and immunoglobulins, it is plausible that WRS and Trp cooperate to regulate the activities of various immune cells^78,79^. Clinically, along with changes in the ratio of serum kynurenine, a metabolite of the IDO pathway, to tryptophan, an imbalance in the expressions of IDO and WRS has been suggested to be associated with autoimmune disorders. Patients with Graves’ disease (GD) show increased kynurenine to Trp ratios and have increased IDO expression in B cells and dendritic cells (DCs), indicating immune suppression; however, GD-derived CD4+ T cells have increased WRS expression and their proliferation was not inhibited by IDO expression in DCs from GD-patients, suggesting the activation of autoreactive T cells^80^. Furthermore, in immune thrombocytopenia, decreased IDO expression and increased WRS expression in CD4+ and CD8+ cells have been proposed to enhance the survival of autoreactive T cells^81^. In rheumatoid arthritis patients, the increased expression of WRS mRNA may be a cause of CD3+ T cell activation (Fig. 3b)^58^.

## Conclusion and future prospects

Beyond its role in protein synthesis, WRS has evolved to play a role in immune regulation. Following an infection, WRS, in combination with WHEP, is immediately secreted by monocytes, the first responder cells, leading to inflammatory reactions that eliminate invading pathogens^20^. Since FL-WRS serves as an endogenous ligand of human TLR2 and TLR4, it is easy to conclude that this protein plays a significant role as an immune activator in various immune responses, including innate immunity, the development of immune cells, and differentiation, as well as adaptive immunity^72,74,78^. Moreover, WRS has simultaneously acquired anti-inflammatory properties through diverse mechanisms such as alternative splicing and proteolytic cleavage^39,45^. WRS is the only ARS whose expression is induced by the IFN-γ produced by innate immune cells and T cells under various immunological contexts. WRS also plays a central regulatory role in IFN-γ-induced anti-angiogenesis and cell survival or death. Since inflammation increases protease activity, the hyper-secretion of FL-WRS results in increased levels of T2-WRS, which suppresses the spread of inflammation through its angiostatic activity and the activation of p53 in the nucleus. FL-WRS, containing the WHEP domain, can also induce a pro-apoptotic state. In addition, during Trp metabolism, WRS participates in the activation and/or inhibition of immune cells, including macrophages, dendritic cells, and T cells, by providing tryptophan-binding activity. By placing a device within the N-terminal extension that can perform two different mechanisms, either activating or inhibiting inflammation, WRS serves as a “yin-yang” modulator of inflammation (Fig. 3).

Sepsis is a syndrome with heterogeneous immunopathology, including acute hyper-inflammation and immunosuppression that cannot overcome nosocomial and opportunistic infections^82^. In sepsis patients who have high WRS levels in the early acute phase, excessive WRS-induced inflammation probably occurs, and antagonizing WRS may be a therapeutic strategy for sepsis; by contrast, in immunocompromised septic patients with monocyte dysfunction, injections of FL-WRS or N154 may serve as an appropriate treatment to boost weakened immunity.

Studies on the function of WRS, not only as a p53 modulator but also an angiostatic agent, suggest that WRS could be a therapeutic target for various diseases, including cancer. Current clinical data examining the expression of WRS in cancer tissues indicate that the role of WRS in tumor biology is not simple and seems to be context-dependent^83^. In several cancers, including gastric adenocarcinoma, colorectal and ovarian cancer, high levels of WRS expression are associated with a favorable prognosis, and WRS was recently included as a predictive biomarker for unnecessary adjuvant chemotherapy after surgery for resectable gastric cancer^46,84,85^. By contrast, in oral squamous cell carcinoma, high levels of WRS are positively correlated with tumor stage, invasion, and depth, and it has been suggested that secreted WRS may induce cancer cell migration^57^. Therefore, how altered WRS expression levels differentially affect the fate of cancer cells and how secreted WRS regulates the cancer microenvironment that contains various cells, including immune cells, are interesting questions. In order for WRS to be meaningful as a prognostic marker, or even as a therapeutic target, further in-depth research needs to be conducted. Until now, in patients with autoimmune diseases, the purpose of WRS overexpression in immune cells has been assumed to be as a Trp repository; however, this notion arises because only certain phenomena have been examined. Therefore, it is very important to examine the mechanisms underlying immune cell proliferation/activation and metabolism in detail.
